# The power of perception: how perceived language policy shapes intergenerational cultural transmission intent

**DOI:** 10.3389/fpsyg.2026.1768026

**Published:** 2026-03-12

**Authors:** Wenjing Wang

**Affiliations:** Department of Chinese and Media, Bozhou University, Bozhou, China

**Keywords:** cultural heritage, ecological systems theory, intergenerational transmission, language vitality, perceived language policy, social cognition

## Abstract

**Introduction:**

Amidst global challenges to linguistic diversity, the intergenerational transmission of local languages is critical for cultural preservation. While public enthusiasm is high, it is unclear how this translates into transmission intention, particularly how macro-level policies shape individual psychology. Existing research often lacks an integrated framework. To address this gap, this study proposes and tests an integrated social-ecological-cognitive model, hypothesizing that perceived language policy, as a macrosystem factor, is the most critical predictor of intergenerational language transmission intention.

**Methods:**

An online survey was conducted across China, yielding 390 valid responses via snowball sampling. The questionnaire measured transmission intention, attitudes, social norms, community engagement, and perceived language policy. Data were analyzed using descriptive statistics, ANOVA, and multiple linear regression.

**Results:**

The results indicated that the ecological model explained 52.9% of the variance in transmission intention, with all dimensions being significant predictors. Notably, perceived language policy emerged as the predictor with the largest standardized coefficient (β = *0.266, p < 0.001*). Additionally, language proficiency was found to be foundational for positive attitudes (*p < 0.001*).

**Discussion:**

In conclusion, intergenerational language transmission intention is shaped by a multi-level ecological system. The public's subjective perception of macro-level institutional support is a primary psychological correlate. For cultural preservation, policies must be made visible and tangible.

## 1 Introduction

As a core symbol of culture and a key component of intangible cultural heritage (Braber and Howard, 2023), the intergenerational transmission of language is not merely a sociological issue; it profoundly reflects a group's core psychological processes, such as cultural identity (Hadirman et al., 2024), collective memory, and future orientation. As language is a primary vehicle for culture, its transmission is central to broader cultural continuity. Amidst the currents of globalization and informatization, linguistic diversity faces unprecedented challenges, with recent interdisciplinary research predicting that over half of the world's endangered languages will become extinct by the end of this century (Bromham et al., 2022). In China, while the promotion of the national standard language has achieved tremendous success, the intergenerational transmission of local languages, as integral parts of Chinese culture, is facing significant challenges (Bilgory-Fazakas and Armon-Lotem, 2025).

In recent years, with a rise in cultural confidence, the public has shown strong identification with the cultural and emotional value of local languages, viewing them as key cultural heritage and a motivation for sustainable protection (Xu et al., 2024). However, can this surging cultural enthusiasm effectively translate into an optimistic outlook for the future of these languages? More importantly, how does the macro-level policy environment influence this intention through individual subjective perception?

Existing research has often focused on single dimensions, such as separately exploring the impact of attitudes or social vitality (Duan, 2022). However, the latest research trends emphasize the necessity of understanding the complex interplay of these factors within an integrated framework. For instance, studies are examining how family language policy, ethnic identity, and cultural practices jointly affect language transmission and maintenance (Fatima and Nadeem, 2025). The insight from these studies is that a unified framework is urgently needed to examine the interaction between macro-level institutional factors and micro-level psychological perceptions. Within this context, the psychological effect of language policy is a particularly crucial yet often overlooked link. While policy serves as a macro-level tool for shaping the social status of a language, its implementation ultimately depends on public perception. Recent studies increasingly highlight that it is an individual's subjective perception of the macro-policy environment, rather than the policy text itself, that is the key psychological variable shaping their language attitudes and behaviors (Pagé and Noels, 2024). This aligns with foundational principles in cognitive and social psychology, which posit that objective reality influences behavior only after being filtered through an individual's subjective construction and interpretation (e.g., the Thomas theorem). It is not the policy-as-written, but the policy-as-perceived that constitutes the psychologically potent reality for individuals. For example, in multilingual settings, learners' perception of institutional support has been shown to significantly predict their language learning motivation and cultural identity (Bilgory-Fazakas and Armon-Lotem, 2025). This perception directly influences individuals' psychological safety, identity, and sense of collective efficacy, which in turn determines their willingness to undertake the responsibility of intergenerational transmission.

Based on this, the present study aims to move beyond the confines of single-factor determinism by drawing on Bronfenbrenner (1979) ecological systems theory to propose an integrated social-ecological cognitive model. This model operationalizes the theory by mapping different psychological and social factors onto nested ecological levels, from individual attitudes to macro-level policies. It is argued that the public's intention for the future transmission of local languages is not dictated by any single factor but rather stems from a comprehensive perception of the health of the entire linguistic ecosystem. This study specifically seeks to determine if “perceived language policy” plays a distinct macro-level predictive role within this system.

To operationalize this model and explore the broader ecological system, this research seeks to answer the following questions:

1) What is the current state of public perception regarding the five key psychological constructs of the linguistic ecosystem?

2) Are there significant differences in perception on these core issues among groups with different linguistic backgrounds (i.e., language proficiency and family language environment)?

3) After controlling for demographic variables, what is the respective psychological predictive power of the five ecological dimensions on the public's future transmission intention, and which dimension serves as the primary statistical predictor?

Theoretically, this study attempts to integrate Bronfenbrenner's ecological systems theory with attitude-behavior theories (e.g., the Theory of Planned Behavior) and apply them to the unique domain of cultural transmission. By constructing and testing a multi-dimensional integrated model, it seeks to reveal the complex psychological mechanisms influencing intergenerational cultural transmission intention, thereby promoting a paradigm shift in related research from a focus on “single psychological variables” to “multi-level ecological systems.”

Practically, the findings of this study will offer valuable insights for language policymakers, educators, and community advocates. If “perceived language policy” can be accurately identified as the core statistical leverage point, it will help direct limited social resources toward areas that can generate the most substantial psychological impact, thus more effectively fostering a sociocultural environment conducive to the healthy transmission of local languages.

## 2 Literature review and theoretical framework

### 2.1 Literature review

The issue of intergenerational language transmission has long been a focal point in sociolinguistics and social psychology. Existing empirical work has largely clustered around micro-level factors, confirming the family as the primary site for language socialization. Recent empirical work has begun to explore these complex dynamics. For instance, qualitative studies with Canadian adults indicate that childhood language experiences significantly shape future transmission intentions (Pagé and Noels, 2024). Similarly, mixed-methods approaches highlight how family dynamics and ethnic identity intersect to influence language maintenance (Fatima and Nadeem, 2025). In the Chinese context, research on Chaoshan families suggests that while local dialects remain pivotal for constructing identity, they face attrition among the young (Fang and Yao, 2025). Likewise, studies on Tibetan families reveal that whilst agency is exerted through religious and educational practices, families must constantly negotiate with broader ideologies (Yao and Nie, 2025). These studies robustly confirm that the “microsystem” is indispensable.

However, a reliance solely on micro-level explanations creates a theoretical gap. As argued in recent work Beyond the Home, “Family Language Policy (FLP) alone cannot account for the various sociopolitical pressures,” characterizing language maintenance as a “collective responsibility” (Inan and Harris, 2025). Critics argue that without macro-level institutional support, family-level efforts are often unsustainable (Fishman, 1991). Empirical evidence supports this limitation; despite positive parental attitudes, immigrant parents often feel “disempowered” when their efforts contradict macro-level national policies (Liu et al., 2024). This highlights the discrepancy between micro-level agency and macro-level constraints.

This suggests a need to shift focus from objective policy texts to how these policies are perceived by individuals. While some studies describe policy merely as contextual background—such as recent reviews on English-medium instruction in China (Liao et al., 2025)—emerging trends emphasize the psychological agency of individuals. As noted in recent scholarship, there is a call to integrate FLP within broader sociopolitical contexts, yet quantitative models explicitly measuring “perceived institutional support” remain rare (Zabrodskaja, 2025a). This study seeks to bridge this divide by integrating the macro-perspective into the psychological modeling of intention. By operationalizing these multi-level factors into a single quantitative model, this research positions “perceived policy” alongside traditional micro-variables. This allows for a statistical comparison of their predictive power, specifically testing the primacy of the macro system, thereby synthesizing the conflicting emphases on “home vs. society” found in current literature.

### 2.2 Theoretical framework

Intergenerational language transmission is fundamentally a behavioral intention. Traditional social psychological models, such as the Theory of Planned Behavior (TPB), explain such intentions through individual-level constructs like attitudes and social norms. However, these models often isolate individual cognition from the broader socio-structural context. This is a significant limitation in language research where macro-level factors like government policy are highly influential. Therefore, this study seeks to bridge this gap by integrating such individual-level psychological mechanisms within a broader ecological framework.

The work of Fishman (1991) established the central importance of intergenerational transmission. From a psychological perspective, this transmission is essentially a form of prosocial behavioral intention. To systematically clarify its influencing factors, this study adopts Bronfenbrenner (1979) ecological systems theory (EST) of human development as its overarching analytical framework. We selected EST primarily to break free from the “laboratory vacuum” of traditional psychological research, emphasizing that individual behavioral intentions are shaped by the combined influence of nested microsystems, mesosystems, exosystems, and macro systems. This framework uniquely suits the current study as it provides a “wide-angle lens” for examining influencing factors from proximal (e.g., family) to distal (e.g., policy) levels, offering a theoretical justification for operationalizing variables such as Perceived Language Policy and Perceived Social Norms alongside individual attitudes.

### 2.3 A social-ecological cognitive model of intergenerational cultural transmission intention

Bronfenbrenner's theory outlines five nested environmental systems: the Microsystem (immediate environment), Mesosystem (connections between microsystems), Exosystem (indirect environments), Macro system (cultural blueprints), and Chronosystem (time). This study's model incorporates constructs corresponding to the first four systems. The Chronosystem, which involves longitudinal change, is beyond the scope of this cross-sectional study, though it is acknowledged that shifts in language policy over time undoubtedly shape current perceptions. By integrating individual cognitive constructs from social psychology into this structure, a more holistic analysis is possible.

Drawing on Bronfenbrenner's ecological systems theory (EST) and integrating research from social psychology on attitude-behavior relationships, the model constructed in this study aims to examine how environmental factors at different nested levels jointly shape the public's intention for the future transmission of local languages.

**Figure 1:**
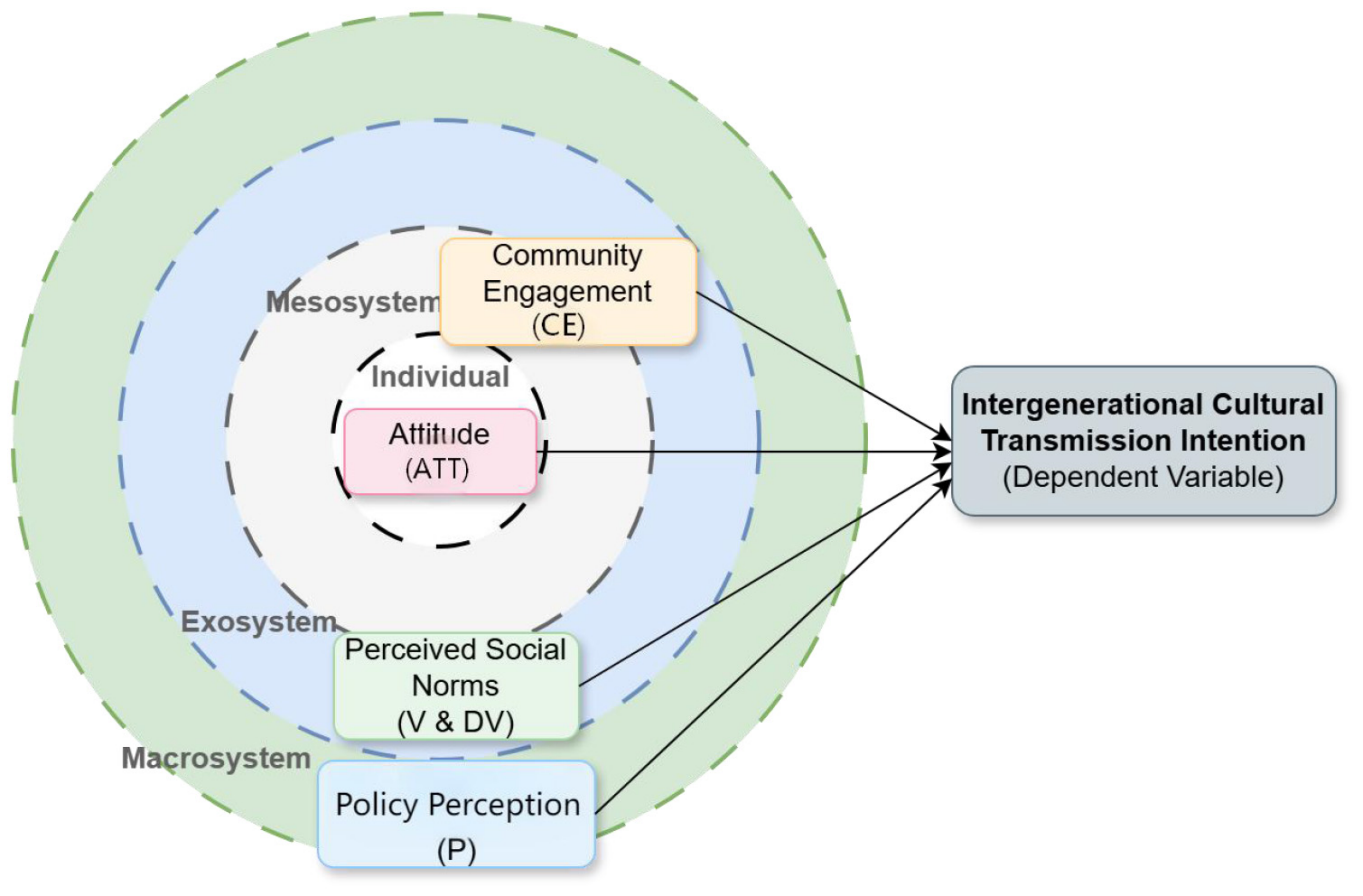
Theoretical model of intergenerational cultural transmission intention based on an ecological systems framework.

As shown in Figure 1, the core predictor variables were classified the core predictor variables into ecological levels and provided a theoretical justification based on their cognitive distance from the individual and their mechanisms of action:

Attitude (ATT) - Individual System: This variable is at the center of the model, representing an individual's internal value judgment of the local language, primarily covering the subjective cognition of its functional value (e.g., commercial and social utility) and identity value. It is the final recipient of all external environmental influences, and its outcome directly affects the individual's intention to transmit the language to the next generation (Pagé and Noels, 2024). While the microsystem traditionally emphasizes social interactions (e.g., within the family), this model operationalizes the psychological outcome of these interactions as an individual-level attitude. The direct influence of the family environment is controlled for separately as a demographic variable.

Community Engagement (CE) - Mesosystem: Classic EST defines the mesosystem as the connections between microsystems (e.g., family, school). This study places “Community Engagement” at this level to emphasize the individual's active interaction and participatory experience with their immediate community (neighborhoods, civil society organizations). It reflects the proximal manifestation of community-level institutional support and cultural vitality, aligning with the role of the community emphasized in ethno linguistic vitality theory (Olko, 2024). Specifically its dimension of institutional support.

Perceived Social Norms (V/DV) – Exosystem: This variable measures the general perception of the language's use in both traditional domains (V) and new digital media domains (DV). This study functionally combine these two types of norms and place them in the exosystem based on the following rationale:

Although V (traditional vitality) is rooted in micro-level interactions (e.g., family), its role in the model is primarily that of a broad social referent (i.e., “what most people are doing”), constituting indirect social pressure and expectations.

DV (digital vitality) represents social norms shaped by indirect environments such as mass media and online platforms (typical exosystems). Therefore, classifying both under the exosystem aims to focus on how broad social reference groups exert an indirect shaping influence on individual transmission intention through normative pressure.

Perceived Language Policy (P) – Macro system: This is the core construct of our study. Policy perception represents the societal ideology and institutional support that influence all inner systems. The word “perception” is emphasized” because it measures the public's subjective experience and psychological expectation of government support in areas like public services, the education system, and cultural funding. Drawing on Spolsky (2004) language policy framework, which involves language practices, beliefs, and management, this construct assesses the public's perception of government management efforts. This perception directly affects the language's social legitimacy and is theoretically posited as a key structural factor in stimulating collective beliefs, such as collective efficacy (Zabrodskaja, 2025b).

Based on the theoretical framework, specifically regarding the role of macro systems, this study puts forth the following central hypothesis:

Hypothesis: Perceived language policy, as a macro system factor, will emerge as the predictor with the largest standardized coefficient for intergenerational language transmission intention, even after controlling for more proximal factors.

## 3 Methods

To address the research questions outlined above and test the proposed social-ecological-cognitive model, the following methods were employed.

### 3.1 Participants

This study employed an online questionnaire survey, using snowball sampling through social networking platforms (WeChat, Weibo). A total of 402 questionnaires were collected. To ensure data quality, several screening criteria were applied: responses with completion times of less than 180 seconds and those that failed an embedded attention-check question were removed. Responses of “Don't know/Prefer not to say” were treated as missing data. This resulted in 390 valid questionnaires and an effective response rate of 97.0%. It should be noted that due to the non-random nature of snowball sampling, the sample may have a self-selection bias, favoring individuals who are more interested in local language protection, have higher education levels, and are more active online. This may have led to a slight overestimation of the overall public transmission intention (the mean of the dependent variable was 4.13). The demographic characteristics of the sample are presented in Table 1.

**Table 1:** Description of sample demographics (*N* = 390).

**Variable**	**Category**	**Frequency (*n*)**	**Percentage (%)**
Age	1.18 and under	8	2.1
	2.19-30	119	30.5
	3.31-45	128	32.8
	4.46-60	130	33.3
	5.61 and over	5	1.3
Local language proficiency	1. Fluent, native-like	259	66.4
	2. Fluent, but with an accent or limited vocabulary	98	25.1
	3. Can hold simple daily conversations	23	5.9
	4. Can mostly understand but cannot speak	7	1.8
	5. Cannot understand at all	3	0.8
Family language background	1. Almost exclusively local language	175	44.9
	2. Mix of local language and mandarin	169	43.3
	3. Almost exclusively mandarin	38	9.7
	4. Other	8	2.1

### 3.2 Instruments

The questionnaire was pilot-tested with a small group (*N* = 30) to ensure item clarity. All core constructs in this study were measured using a 5-point Likert scale. The full questionnaire is provided in Appendix A. Specific information, reliability indicators, and sample items for each scale are shown in Table 2.

**Table 2:** Overview of measurement instruments for core variables.

**Construct**	**Source and references**	**Sample item**	**Cronbach's α**
Transmission Intention (INT) and Attitude (ATT)	Items were adapted from Gardner and Lambert (1972) social psychological paradigm; incorporates considerations of instrumental value from the Theory of Planned Behavior (Ajzen, 1991).	“I hope my next generation can also learn and use our local language.”	0.92 and 0.89
Language Vitality (V) and Digital Language Vitality (DV)	Items were adapted from Cialdini et al. (1990) model of social influence regarding perceptions of language vitality; and the latest operationalization by Grose-Hodge et al. (2025).	“I often see people communicating in our local language on social media.”	0.87 and 0.91
Community Engagement (CE)	Items were adapted from McMillan and Chavis (1986) Sense of Community Scale.	“I often participate in community activities related to our local language and culture.”	0.88
Perceived Language Policy (P)	Items were adapted from Spolsky (2004) theory of language management; references measures of perceived support from (Pagé and Noels 2024).	“I can feel that the government is making efforts to protect our local language.”	0.93

### 3.3 Data processing and analysis

Data were processed and analyzed using Python (3.9) and its scientific computing libraries (Pandas, NumPy, SciPy, Statsmodels). The main analysis steps were as follows:

Data Preprocessing: Raw data were cleaned, which included handling invalid responses, addressing missing values, and computing variables (calculating the mean for each scale).

Common Method Bias Test: As all data were self-reported, we used Harman's single-factor test to check for common method bias. The results showed that the un rotated factor analysis extracted multiple factors with eigenvalues greater than 1, and the first factor explained 34.2% of the variance (below the 40% critical threshold), indicating that common method bias was not a serious issue in this study. Reliability for all scales was high (Cronbach's α > 0.87, see Table 2). While a formal Confirmatory Factor Analysis (CFA) was not conducted, which is a limitation, the model's theoretically consistent results provide initial support for the constructs' validity.

Descriptive Statistics and Difference Testing: Means and standard deviations were calculated for all core variables. One-way ANOVA was used to test for significant differences in scores on the core variables among different demographic groups.

Predictive Model Construction: A multiple linear regression analysis was conducted with the five ecological dimension means and demographic variables as independent variables, and transmission intention as the dependent variable. The model also included Family Language Background as a covariate to control for its strong influence. To avoid multicollinearity, the independent variables were checked, and the results showed that the Variance Inflation Factor (VIF) for all variables was less than 3, indicating no serious multicollinearity in the model. Assumption checks for regression (e.g., residual diagnostics) were conducted and indicated that the assumptions were adequately met.

A comprehensive assessment of data normality was also performed for the core constructs. Descriptive statistics (skewness and kurtosis) and formal normality tests (Shapiro-Wilk) were computed. As detailed in Appendix B (see Table B1), the Shapiro-Wilk tests were statistically significant for all constructs (*p* < 0.001), indicating that the strict assumption of normality is not met. However, the absolute values for skew ness and kurtosis all fell well within commonly accepted thresholds, and visual inspection of the Q-Q plots (see Figure B1 in Appendix B) confirmed that the deviations were mild. Given the large sample size (*N* = 390) and the inherent robustness of multiple linear regression to mild non-normality, proceeding with parametric statistical analyses was deemed methodologically appropriate despite the statistically significant Shapiro-Wilk results.

## 4 Results

### 4.1 Descriptive Statistics

The means for the six core constructs are shown in Table 3.

**Table 3:** Descriptive statistics for core constructs (*N* = 390).

**Construct**	**Mean (*M*)**	**Standard deviation (SD)**	**Overall rating**
Transmission Intention (INT)	4.13	0.81	Very strong
Attitude (ATT)	4.08	0.70	Highly positive
Traditional Language Vitality (V)	3.80	0.69	Moderately high
Digital Language Vitality (DV)	3.58	0.83	Quite active
Community Engagement (CE)	3.18	0.85	Moderate
Perceived Language Policy (P)	3.01	0.96	Neutral to weak

The means indicate that the public's “future transmission intention” (*M* = *4.13*) and “attitude” (*M* = *4.08*) are at very high levels, demonstrating strong subjective willingness and a solid popular foundation. However, perceptions of the real-world context are relatively more conservative, especially for “community engagement” (*M* = *3.18*) and “perceived language policy” (*M* = *3.01*), which were rated the lowest, hovering around a neutral level.

**Figure 2:**
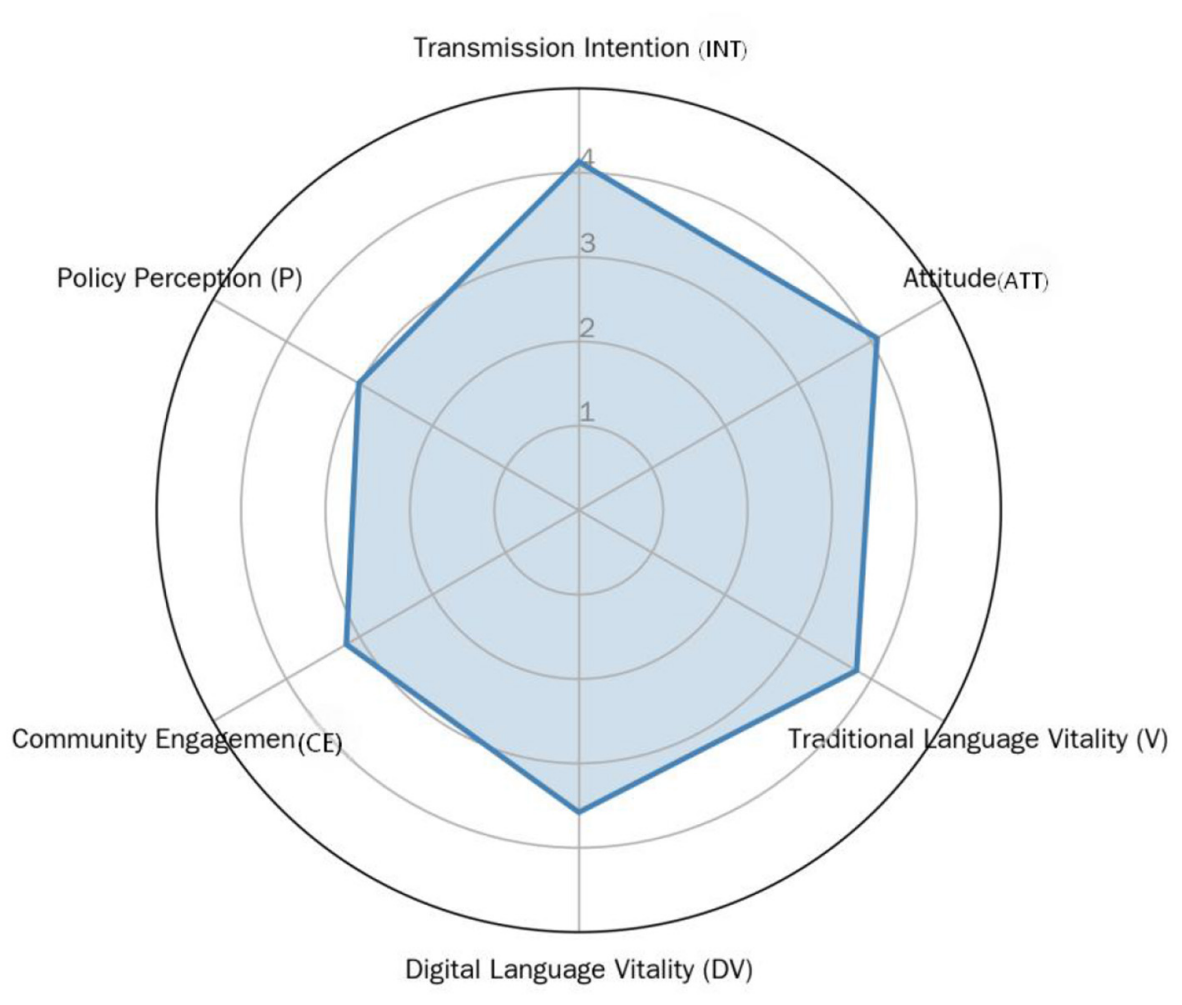
Radar plot of core construct means.

To more intuitively display the tension between this “high intention” and “weak perception,” the means were plotted of the six core constructs on a radar chart (Figure 2).

As shown in Figure 2, the upper-right portion (representing “Transmission Intention” and “Individual Attitude”) is prominently extended, while the lower-left portion (representing “Community Engagement” and “Policy Perception”) is relatively retracted. This imbalance in shape visually confirms the core contradiction revealed by the descriptive statistics: respondents possess strong internal motivation for transmission, but the perceived external environmental support (especially at the community and policy levels) is relatively lacking.

### 4.2 Group difference analysis (ANOVA)

Differences in perception were further examined in perception on several core issues among groups with different demographic backgrounds (Table 4).

**Table 4:** Summary of ANOVA results for group differences.

**Test**	**Grouping variable**	**Dependent variable**	***F***	***p***	**Result**
Test 1	Age	Digital Language Vitality (DV)	F _(3, 386)_ = 0.20	0.940	No significant difference
Test 2	Local language proficiency	Attitude (ATT)	F_(4, 385)_ = 11.27	<0.001	Significant difference exists
Test 3	Family language	Future Transmission Intention (INT)	F_(2, 387)_ = 14.54	<0.001	Significant difference exists

Interpretation of Results:

Test 1: A “Cross-Generational Consensus” on Language Vitality. The results show no statistically significant difference in the perception of digital language vitality across different age groups [*F*_(3, 386)_ = *0.20, p* = *0.940*]. This finding suggests that the social representation of the local language's role in the digital era may be homogenizing across generations, forming a widespread consensus that transcends age.

Test 2: The “Value-Empowering” Effect of Language Proficiency.

The ANOVA results indicate a statistically significant difference in language attitudes (perceived value) toward the local dialect among groups with varying proficiency levels [*F*_(4, 385)_ = *11.27, p*<*0.001*]. This result reveals a “value-empowering” effect of language ability: the stronger an individual's proficiency, the more positively they perceive the language's value and identity functions. A *post-hoc* analysis using Tukey's HSD test further clarified this effect, revealing that individuals with ‘fluent, native-like' proficiency reported significantly higher positive attitudes (M = 4.25, SD = 0.58) compared to those who could ‘mostly understand but cannot speak' (M = 3.63, SD = 0.70), with *p*<*0.001*. Similarly, those who could ‘communicate fluently' also held significantly more positive attitudes than the passive understanding group (M = 4.14 vs. M = 3.63, *p*<*0.001*).

Test 3: The Microsystem (Family) as the “Bedrock” of Transmission.

The results show that one's family language background has a significant impact on their future transmission intention [*F*_(2, 387)_ = *14.54, p*<*0.001*]. *Post-hoc* tests further revealed a clear gradient. Individuals who grew up in ‘almost exclusively local language' households reported the highest transmission intentions (M = 4.41, SD = 0.61), which was significantly higher than the ‘almost exclusively Mandarin' group (M = 3.89, SD = 0.80, *p*<*0.001*). Notably, the ‘mixed use' group (M = 4.20, SD = 0.61) also showed significantly higher intentions than the Mandarin-only group (*p*<*0.001*), providing strong empirical support for the notion that “the family is the primary front for language transmission.”

### 4.3 Relationships among core constructs and prediction of transmission intention

#### 4.3.1 Correlation analysis among core constructs

**Figure 3:**
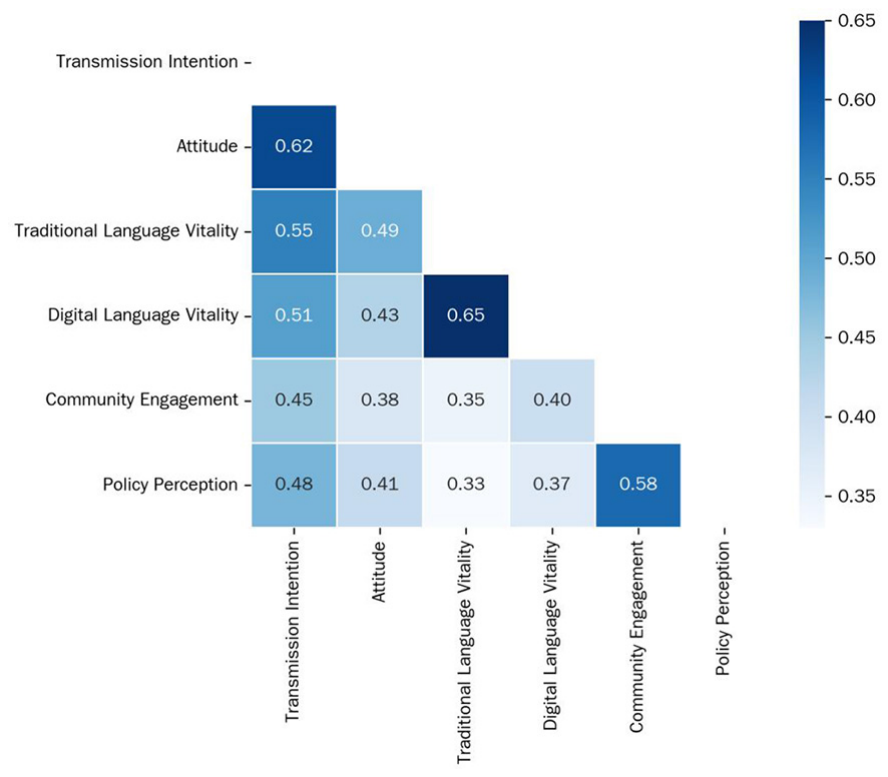
Heatmap of correlations among core constructs.

Before building the predictive model, we first examined the pairwise relationships among the six core constructs. The Pearson correlation coefficient was calculated (r) for each pair of variables and visualized the results in a heat map (Figure 3).

The heat map shows that all five ecological dimensions are statistically significantly and positively correlated with ‘Transmission Intention.' Notably, ‘Attitude' has the strongest correlation with ‘Transmission Intention' (*r* = 0.62), while ‘Perceived Language Policy' also shows a strong, significant correlation (*r* = 0.48), providing strong preliminary evidence for this variable's importance in the subsequent regression analysis.

#### 4.3.2 Multiple regression analysis of transmission intention

To further test the predictive power of the five ecological dimensions and demographic variables on transmission intention, a multiple linear regression model was constructed. The overall model fit is summarized in Table 5.

**Table 5:** Overview of the multiple linear regression model for future transmission intention.

**Model fit metric**	**Value**
Valid Sample Size (*N*)	390
R-squared (*R*^2^)	0.536
Adjusted R-squared	0.529
*F*-statistic	62.93
Model significance [Prob (*F*-statistic)]	<0.001

The overall model fit is excellent. The adjusted R^2^ of 0.529 means that the independent variables in the model can jointly explain 52.9% of the total variance in the dependent variable, transmission intention.

**Figure 4:**
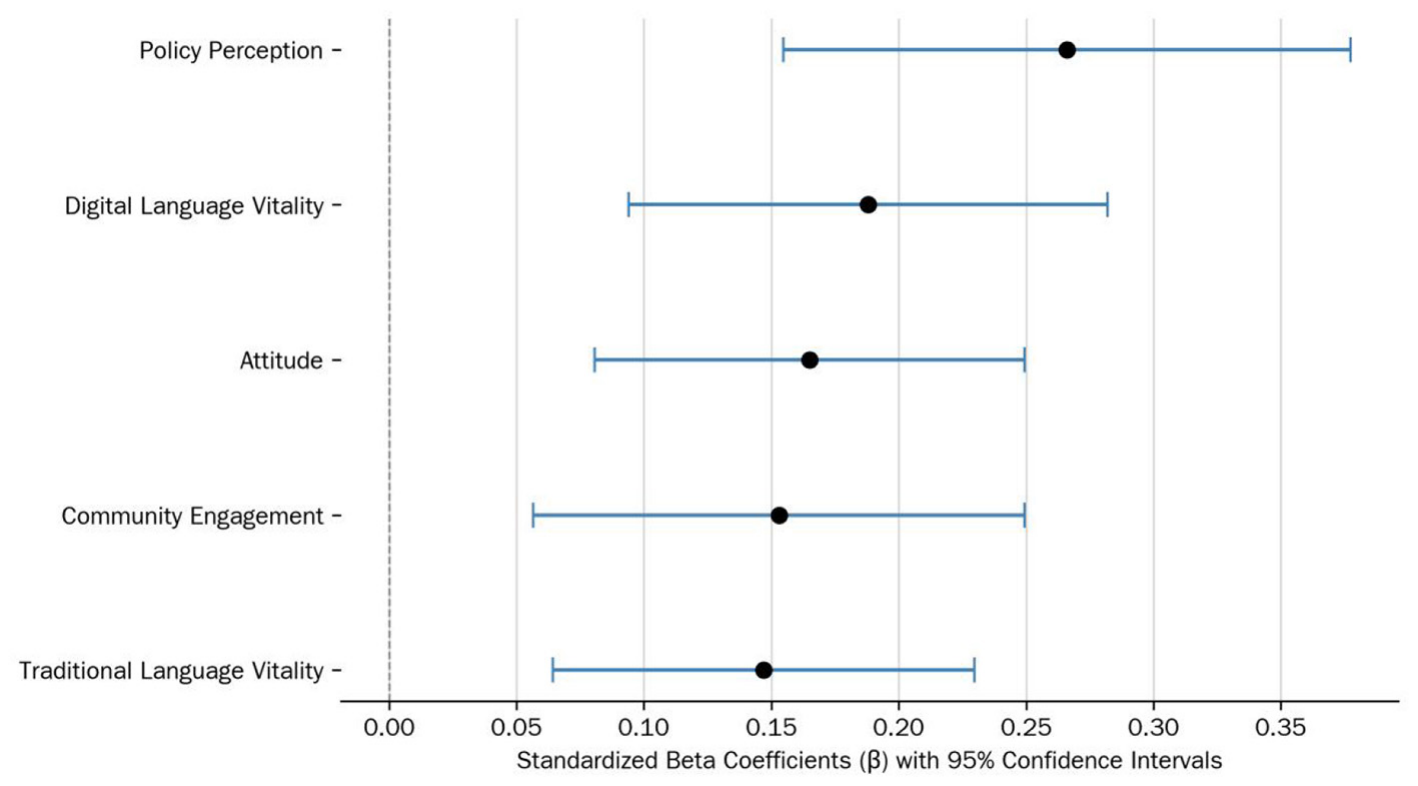
Standardized beta coefficients for predicting future transmission intention (with 95% confidence intervals).

The specific model coefficients are shown in Table 6. To visually compare the predictive strength of each significant variable, their standardized regression coefficients have been plotted (Beta) in Figure 4.

**Table 6:** Detailed coefficients of the multiple linear regression model.

**Predictor variable**	***B***	***SE***	**β**	***t***	***p***
(Intercept)	0.148	0.134		1.107	0.269
Perceived Language Policy (P)	0.224	0.048	0.266	4.688	<0.001^***^
Digital Language Vitality (DV)	0.183	0.047	0.188	3.920	<0.001^***^
Attitude (ATT)	0.189	0.049	0.165	3.832	<0.001^***^
Community Engagement (CE)	0.144	0.046	0.153	3.112	0.002^**^
Language Vitality (V)	0.170	0.049	0.147	3.483	0.001^**^
Age	−0.048	0.027	−0.076	−1.570	0.117
Local Language Proficiency	0.004	0.041	0.005	0.094	0.925
Family Language Background	0.035	0.038	0.041	0.921	0.358

The dependent variable is Transmission Intention (INT). Model controls for Age and Family Language Background ^**^p < 0.01, ^***^p < 0.001.

#### 4.3.3 Conclusions from regression analysis

The overall validity of the ecological systems model is confirmed. The regression model shows that the five core ecological dimensions—individual attitude (ATT), community engagement (CE), traditional language vitality (V), digital language vitality (DV), and perceived language policy (P)—all exhibit statistically significant positive predictive power on future transmission intention (all *p* < 0.01). Figure 4 visually confirms this, as the 95% confidence intervals for all five variables do not include zero, strongly supporting the social-ecological cognitive model of intergenerational transmission proposed in this study.

The prominent statistical role of macro-level policy perception. Among all significant predictors, “Perceived Language Policy” has the largest standardized coefficient (β = *0.266*), suggesting its notable statistical association with the outcome within this specific model. This finding highlights that a perceptible macro-policy environment is the most critical psychological lever for influencing public transmission intention.

Indirect influence and mediating effects of personal background factors. The model shows that after including the ecological system variables, the direct predictive effects of the demographic variables—“Age” (*p* = *0.117*) and “Local Language Proficiency” (*p* = *0.925*)—are no longer significant. This result reveals the overwhelming influence of ecological perceptions: for the formation of transmission intention, the public's collective assessment of the multi-level environment is more important than isolated individual background differences. This is particularly insightful for “Local Language Proficiency,” as it confirms that its influence does not disappear but is fully mediated by the shaping of the more proximal “Attitude,” playing an irreplaceable “cornerstone” role rather than a direct driving one.

## 5 Discussion

This study, based on Bronfenbrenner (1979) ecological systems theory, has systematically examined the multi-level psychological factors influencing intergenerational cultural transmission intention through an empirical analysis of an online sample.

### 5.1 From single attitudes to an ecological system: an integrated prediction of transmission intention by multi-level environments

The core theoretical contribution of this study is the confirmation of a “five-pillar” ecological systems model that influences transmission intention. The regression analysis shows that the psychological constructs of the five ecological dimensions—individual attitude, social norms, community engagement, language vitality, and perceived language policy—are all significant predictors of transmission intention.

This finding is a powerful extension of the classic Theory of Planned Behavior (TPB) in the domain of cultural transmission. Our research demonstrates that cultural transmission intention, as a complex collective behavioral intention, requires predictors that span multiple systems from micro to macro levels. The public's will to transmit is not an isolated individual choice but rather the result of a comprehensive psychological assessment of the health of a multi-level ecological environment. This multi-dimensional psychological systems logic also serves as a reminder that any “single-pronged” cultural preservation effort may have limited effect. Indeed, the moderate to strong correlations observed between the ecological factors in the model (Figure 3), while not posing a multicollinearity threat, empirically support this systemic view. They suggest that these environmental layers are not isolated but are dynamically interconnected, where a positive perception in one area (e.g., policy) likely co-occurs with and reinforces positive perceptions in others (e.g., social norms). Future cultural preservation work must adopt a systematic, multi-sector, and collaborative ecological governance approach (Han, 2023). This systemic finding not only aligns with recent qualitative calls for an ecological governance approach (Han, 2023), but also empirically extends micro-level studies that focused primarily on family language policy (Fatima and Nadeem, 2025) by demonstrating the quantifiable and superior predictive power of the macrosystem.

### 5.2 The primacy of the macro system: a theoretically inferred core psychological driver

Within the “five-pillar” model, “perceived language policy” from the macro system stands out, exhibiting the largest (β = *0.266*)in the model. This finding is the study's most significant contribution to policy psychology, lending support to our core hypothesis. This result indicates that, within this model, the magnitude of the standardized coefficient for the macro system factor is larger than the coefficients for other more proximal factors, such as personal attitude and community factors. This finding of macro-level perceptual primacy resonates with research in other domains. For instance, in public health, perceived governmental support for health initiatives is a powerful predictor of public health-compliance behaviors, often more so than individual health beliefs. Similarly, in environmental psychology, the public's perception of strong pro-environmental government policies is a key driver of household recycling intentions, beyond personal environmental attitudes. This resonates with the view of social policy as a macro-level social determinant of mental health (Kirkbride et al., 2024).

Crucially, this empirical evidence resolves the theoretical tension highlighted earlier in the literature review between micro-agency and macro-structure. While recent qualitative studies emphasize the family as the primary site of socialization (Pagé and Noels, 2024), the present results align with the critique that family efforts are often “disempowered” when they contradict macro-level constraints (Liu et al., 2024). The primacy of perceived policy in this study confirms Inan and Harris (2025) assertion that language maintenance must be viewed as a “collective responsibility” supported by sociopolitical structures, rather than a private burden.

This finding strongly supports the study's theoretical assumptions. Although the cross-sectional design limits our ability to formally test for mediation, based on existing literature in social psychology and language vitality theory, it is speculated that the powerful predictive ability of ‘perceived language policy' is likely realized through two key psychological mechanisms:

1) Shaping legitimate status and psychological safety: positive policy signals are the strongest indicators of a local language's “social prestige” and “legitimacy in public use.” When the public perceives proactive government measures, it significantly reduces the psychological risks and identity anxiety associated with using the local language in public spaces.

2) Bolstering collective efficacy: collective efficacy is a group's belief in its ability to accomplish long-term, ambitious tasks together. It is speculated that support from language policy is viewed by the public as “institutional endorsement” and an “external resource guarantee.” This powerfully stimulates the public's collective belief that “we can succeed,” thereby successfully converting “high expectations” for transmission into a “strong intention.”

Therefore, the “perceived language policy” measured in this study serves as a significant channel for the government to send signals of psychological safety, enhance the social status of the language, and thereby catalyze the positive functioning of the entire ecosystem.

### 5.3 Language proficiency as the bedrock of attitude formation: from “usable” to “valued”

A key finding of this study is that an individual's proficiency in their local language is significantly and positively correlated with their attitudes toward its instrumental and identity value (*F*_(4, 385)_ = *11.27, p*<*0.001*). This result clearly indicates that language ability is a key correlate in shaping positive attitudes, thereby providing strong empirical support for the necessity of language education.

The underlying psychological mechanism of this finding may follow a value transformation path from “usable” to “useful,” and finally to “valued.” When individuals can proficiently use a language, they are more likely to experience its instrumental value (usable, useful) in real social or economic activities. It is the realization of this utilitarian value that further strengthens their identification with and pride in the identity value the language carries (valued). Therefore, relying solely on emotional cultural promotion may not be sufficient to effectively enhance public recognition of a language's value. Instead, practical language education and the creation of frequent usage contexts are the most solid foundations for cultivating positive attitudes. This finding also perfectly explains why the direct predictive effect of language proficiency became non-significant in the final regression model: its influence was likely integrated into the predictive system by shaping the more proximal psychological variable of “Attitude,” where it plays an irreplaceable “cornerstone” role.

### 5.4 Limitations and future directions

The study's sampling method presents limitations. The use of snowball sampling via social media may have introduced a self-selection bias favoring individuals more interested in language issues, potentially inflating intention scores. Moreover, although the online survey was distributed broadly, its precise geographic distribution was not controlled. Future research could benefit from quota sampling to ensure broader geographic representativeness.

A further limitation is the study's cross-sectional design, which inherently restricts our ability to draw strictly causal inferences. Although regression analysis allows us to identify significant associations, it is critical to note that terms like “predictor” or “influence” used throughout this paper refer to statistical relationships rather than proven causal effects; future longitudinal studies are needed to clearly establish directionality. Another limitation is that, while this paper theoretically proposes that ‘collective efficacy' is the core engine through which macro-policy influences individual intention, this variable could not be included in the empirical model for validation. As hypothesized in the theoretical framework, collective efficacy is likely the crucial psychological mechanism linking policy perception to behavioral intent. Therefore, the primary task and highest value for future research is to formally and rigorously test the mediating mechanism through which ‘perceived language policy' influences ‘transmission intention' by enhancing ‘collective efficacy,' using methods such as Structural Equation Modeling (SEM) or longitudinal designs.

A final limitation is found in the statistical analysis itself. While this study identified the predictor with the largest standardized coefficient, it followed the common convention of not employing more advanced statistical methods to partition variance among inter correlated predictors. Future research could utilize relative importance analyses (such as dominance analysis or relative weights analysis) to more robustly determine the unique and shared contributions of each ecological factor, offering a more definitive conclusion on their relative importance.

## 6 Conclusion and policy implications

By constructing and testing a social-ecological cognitive model to predict intergenerational cultural transmission intention, this study concludes that transmission intention stems from a healthy ecological cognitive system. This system is jointly constituted by individual attitudes, social norms, community engagement, and perceived language policy, demonstrating that cultural transmission intention is the result of the combined effects of multi-level environmental factors. Within this system, macro-level policy perception emerges as the predictor with the largest standardized coefficient. Among all predictors, perceptible support from language policy is the strongest predictor of public transmission will (β = *0.266*), with an influence that surpasses even proximal personal attitudes. Furthermore, language proficiency is the cornerstone of attitude formation. An individual's proficiency in their local language significantly predicts their attitude toward it. The stronger the proficiency, the more an individual perceives the language's practical and identity value, thus forming a more solid motivation for transmission.

Based on these conclusions, we propose that language policy should shift from “symbolic” to “perceptible” to strengthen “visible support.” Based on the central finding that perceived language policy had the largest standardized coefficient in the regression model (β = 0.266), local governments should move beyond the static preservation of dialects as “cultural heritage” and instead embrace their dynamic utilization as a “living resource.” It is recommended moderately introducing dialect options in public services (e.g., transportation, healthcare), supporting high-quality dialect programming on local public media, and systematically integrating local language and culture into the curriculum of primary and secondary schools (Ma et al., 2024). These tangible actions are key strategies for enhancing the language's social status. Simultaneously, “linguistic proficiency” should be prioritized as a core focus. Given that our ANOVA results empirically established language proficiency as a prerequisite for shaping positive attitudes (F (4, 385) = 11.27, p <0.001), special funds should be established for immersive language programs, digital learning tools, and community salons. This approach should be underpinned by an “ecological governance” mindset that empowers rather than replaces civil society. In line with the validity of our multi-level ecological model, the government's role should be that of a “platform builder” and an “enabler,” providing targeted funding to stimulate endogenous motivation and foster a vibrant linguistic ecosystem (Wang, 2020).

## Data Availability

The original contributions presented in the study are included in the article/Supplementary material, further inquiries can be directed to the corresponding author/s.

## References

[B1] Ajzen I. (1991). The theory of planned behavior. Organ. Behav. Hum. Decis. Process. 50, 179-211. doi: 10.1016/0749-5978(91)90020-T

[B2] Bilgory-Fazakas O. Armon-Lotem S. (2025). Resilient heritage language maintenance: the interplay of family, culture, and pragmatic choices. Front. Psychol. 16:1550704. doi: 10.3389/fpsyg.2025.1550704 40276672 PMC12018381

[B3] Braber N. Howard V. (2023). Safeguarding language as intangible cultural heritage. Int. J. Intang.Herit. 18, 146-158. doi: 10.35638/ijih.2023.18.0.010

[B4] Bromham L. Dinnage R. Skirgård H. Ritchie A. Cardillo M. Meakins F. . (2022). Global predictors of language endangerment and the future of linguistic diversity. Nat. ecol. Evol. 6, 163–173. doi: 10.1038/s41559-021-01604-y 34916621 PMC8825282

[B5] Bronfenbrenner U. (1979). The Ecology of Human Development: Experiments by Nature and Design (Vol. 352). Cambridge, MA: Harvard University Press. doi: 10.4159/9780674028845

[B6] Cialdini R. B. Reno R. R. Kallgren C. A. (1990). A focus theory of normative conduct: recycling the concept of norms to reduce littering in public places. J. personal. social psychol. 58:1015. doi: 10.1037/0022-3514.58.6.1015

[B7] Duan F. (ed.). (2022). “The ethnolinguistic vitality of Naxi language in Jinshan Bai minority township of Lijiang City,” in 4th International Seminar on Education Research and Social Science (ISERSS 2021) (Dordrecht: Atlantis Press), 191–195. doi: 10.2991/assehr.k.220107.036

[B8] Fang F. Yao X. (2025). Intergenerational transmission and multilingual dynamics: exploring language policies in chaoshan families through a contextual lens. Curr. issues lang. plan. 26, 234–253. doi: 10.1080/14664208.2024.2410517

[B9] Fatima S. Nadeem M. U. (2025). Family language policy and heritage language transmission in Pakistan—the intersection of family dynamics, ethnic identity and cultural practices on language proficiency and maintenance. Front. Psychol. 16:e1560755. doi: 10.3389/fpsyg.2025.1560755 PMC11922832 40115290

[B10] Fishman J. A. (1991). Reversing Language Shift: Theoretical and Empirical Foundations of Assistance to Threatened Languages (vol. 76). Bristol, UK: Multilingual Matters. doi: 10.2307/jj.33169466

[B11] Gardner R. C. Lambert W. E. (1972). Attitudes and Motivation in Second-Language Learning. London, England: Edward Arnold.

[B12] Grose-Hodge M. Dabrowska E. Divjak D. (2025). Bilingual acquisition during school years: predictors of achievement in the societal and heritage language. Front. Lang. Sci. 3:1419563. doi: 10.3389/flang.2024.1419563

[B13] Hadirman H. Hardin H. Musafar M. Ardianto A. (2024). Lokalitas yang terabaikan: menggali konektivitas bahasa dan budaya dalam praktik perladangan suku muna melalui pendekatan ethnolinguistik. Jurnal Ilmiah Ilmu Sosial 10, 139-156. doi: 10.23887/jiis.v10i2.81621

[B14] Han Q. (2023). The preservation and transmission of dialect culture in the context of language ecology. J. Human.Arts Soc. Sci. 7, 1599-1603. doi: 10.26855/jhass.2023.08.022

[B15] Inan S. Harris Y. R. (2025). Beyond the home: rethinking heritage language maintenance as a collective responsibility. Front. Psychol. 16:1616510. doi: 10.3389/fpsyg.2025.1616510 40557361 PMC12185927

[B16] Kirkbride J. B. Anglin D. M. Colman I. Dykxhoorn J. Jones P. B. Patalay P. . (2024). The social determinants of mental health and disorder: evidence, prevention and recommendations. World Psychiatry 23, 58–90. doi: 10.1002/wps.21160 38214615 PMC10786006

[B17] Liao S. J. Zhang L. J. May S. (2025). English-medium instruction (EMI) language policy and implementation in China's higher education system: growth, challenges, opportunities, solutions, and future directions. Curr. Issues Lang. Plan. 26, 1-20. doi: 10.1080/14664208.2025.2453263

[B18] Liu Y. Guo S. Gao X. (2024). Coping with national language policy shift: voices of chinese immigrant parents in an irish county town. Br. J. Educ. Stud. 72, 457–481. doi: 10.1080/00071005.2024.2309604

[B19] Ma J. Zhong Y. Zhao M. (2024). Meaning and Path: Research on the Integration of Dialect Cultural Resources into Civic and Political Education in Colleges and Universities. Ontario, ON: Clausius Scientific Press

[B20] McMillan D. W. Chavis D. M. (1986). Sense of community: a definition and theory. J. commun. Psychol. 14, 6-23. doi.org/10.1002/1520-6629

[B21] Olko J. (2024). 7. Ethnolinguistic vitality, multilingual communication and speakers of contested languages1. *Res. Handbook Commun. Prejudice* 104. doi: 10.4337/9781802209662.00014

[B22] Pagé L. L. Noels K. A. (2024). Family language policy retention across generations: childhood language policies, multilingualism experiences, and future language policies in multilingual emerging Canadian adults. Front. Psychol. 15:1394027. doi: 10.3389/fpsyg.2024.1394027 39323582 PMC11422779

[B23] Spolsky B. (2004).*Language Policy*. Cambridge, England: Cambridge university press.

[B24] Wang H. (ed.). (2020). “Inheritance and development of Chinese dialect culture,” in 6th International Conference on Education, Language, Art and Inter-cultural Communication (ICELAIC 2019) (Dordrecht: Atlantis Press), 924–926. doi: 10.2991/assehr.k.191217.271

[B25] Xu J. Zhou C. Liu H. (2024). Cultural heritage as a key motivation for sustainable language protection: a case study of the suzhou dialect protection project. J. Multiling. Multicult. Dev. 45, 1-18. doi: 10.1080/01434632.2024.2317351

[B26] Yao J. Nie P. (2025). Beyond binary shifts: an ethnographic family language policy study of three generations in a Tibetan household. Curr. Issues Lang. Plan. 26, 1-20. doi: 10.1080/14664208.2025.2545055

[B27] Zabrodskaja A. (2025a). Across languages and borders: empirical advances in family language policy research. Languages 10:142. doi: 10.3390/languages10060142

[B28] Zabrodskaja A. (2025b). Introduction to the special issue ‘exploring language dynamics across (migrant) communities'. J. Multiling. Multicult. Dev. 46, 1-12. doi: 10.1080/01434632.2025.2509790

