# Supplementary material for: The power of perception: how perceived language policy shapes intergenerational cultural transmission intent
Source: Front Psychol. 2026 Mar 12;17:1768026. doi: 10.3389/fpsyg.2026.1768026 (PMC13017853; doi:10.3389/fpsyg.2026.1768026)
Supplement: Supplementary file 1 [file Data_Sheet_1.docx]

### **Appendix A: Survey Questionnaire**

**Introduction**

Dear Participant,

Greetings!

I am an academic researcher dedicated to studying dedicated to studying the transmission and development of local language and culture. This survey aims to gain a deeper understanding of the current vitality, digital ecosystem, policy support, and community engagement related to the language in your region, as well as your perceptions of its value and your hopes for its future.

**Before you begin, please be aware of the following:**

**Anonymity:** This survey is completely anonymous. All data will be used solely for academic research, and your personal privacy will be strictly protected.

**Voluntary Participation:** Your participation is entirely voluntary. You may stop or withdraw from the survey at any time.

**Estimated Time:** Completing this questionnaire will take approximately 15-20 minutes.

Your honest responses are crucial to this research. If you have read and understood the information above and agree to participate in this survey, please select “Agree and Start Survey.”

**Part I: Personal Background and Language Identity**

**1. What city are you in?** __________

**2. What is your age group?**
18 and under
19-30
31-45
46-60
61 and over

**3. What is your personal proficiency in the local language?**
A. Native-like fluency
B. Can communicate fluently
C. Can hold simple conversations
D. Can understand, but can’t really speak it
E. Cannot speak or understand it at all

**4. Growing up, what language was primarily used for communication in your family?**
A. Almost exclusively the local language
B. A mix of the local language and Mandarin
C. Almost exclusively Mandarin
D. Other

**Part II: Your Views and Feelings About the Local Language (Attitude - ATT)**

Please rate your agreement with the following statements based on your personal feelings on a 5-point scale from (1) Strongly Disagree to (5) Strongly Agree.

| Item | Statement |
| --- | --- |
| ATT1 | I believe that knowing the local language is helpful for finding a job or doing business locally. |
| ATT2 | I think the local language, as a cultural resource, can bring economic benefits to local tourism and creative industries. |
| ATT3 | Speaking the local language helps build rapport and gain social acceptance more quickly in this area. |
| ATT4 | I believe our local language is an important symbol of identity that sets us apart from people in other regions. |
| ATT5 | I feel confident and comfortable using the local language, even when everyone around me is speaking Mandarin. |

**Part III: The Current Status of Your Local Language (Traditional Language Vitality - V)**

Based on your general observations of your city, please rate how common the following phenomena are on a 5-point scale from (1) Very Uncommon to (5) Very Common.

| Item | Statement |
| --- | --- |
| V1 | In the community, grandparents/parents speak the local language to their grandchildren/children. |
| V2 | Children and teenagers use the local language to play and interact with each other. |
| V3 | In daily life settings like farmers’ markets and restaurants, people primarily communicate in the local language. |
| V4 | At local festivals, ceremonies, weddings, and other traditional events, the local language is the main language of communication. |
| V5 | Local TV or radio stations have high-quality programs in the local language. |
| V6 | It is possible to find books or educational materials written in the local language. |

**Part IV: Perceived Government Support (Perceived Language Policy - P)**

Based on your observations, please rate the level of support from the local government in the following areas on a 5-point scale from (1) Very Weak Support to (5) Very Strong Support.

| Item | Statement |
| --- | --- |
| P1 | The government uses the local language in public transportation systems (e.g., bus/subway announcements). |
| P2 | Government public service centers (e.g., administrative halls) offer services in the local language. |
| P3 | The government supports the offering of local language interest classes or local culture courses in primary and secondary schools. |
| P4 | The government provides funding or venues for artistic forms like local language theater, folk arts, and songs. |
| P5 | The government supports the inclusion of the local language and its related culture on the intangible cultural heritage list for protection. |

**Part V: Community and Grassroots Cultural Activities (Community Engagement - CE)**

Based on your impressions, please rate the level of activity from local community and grassroots organizations in the following areas on a 5-point scale from (1) Very Low Activity to (5) Very High Activity.

| Item | Statement |
| --- | --- |
| CE1 | Community or grassroots groups spontaneously organize “language corners,” storytelling sessions, or reading clubs. |
| CE2 | There are civil society groups or volunteer organizations dedicated to documenting, researching, or promoting the local language. |
| CE3 | Local artists and cultural creators actively use the local language in their work (e.g., music, theater, literature). |
| CE4 | Local businesses and brands consciously use elements of the local language in their advertising or product naming. |
| CE5 | There are many creative products on the market with a local language theme (e.g., T-shirts, phone cases, sticker packs). |

**Part VI: The Use of the Local Language in Technology and Online (Digital Language Vitality - DV)**

Based on your observations, please rate the prevalence of the local language in digital and technological domains on a 5-point scale from (1) Very Uncommon to (5) Very Common.

| Item | Statement |
| --- | --- |
| DV1 | When chatting on WeChat or posting on social media, people use local language vocabulary or puns based on it. |
| DV2 | It is easy to type unique words and characters of the local language using standard input methods. |
| DV3 | On platforms like Douyin (TikTok) and Bilibili, there is a large amount of high-quality short video or live stream content created in the local language. |
| DV4 | There are dedicated online communities or apps for creating and sharing local language sticker packs, podcasts, or online dictionaries. |

**Attention Check Item**

**AC1. [Attention Check]** To ensure the quality of our data, please select the “Agree” option for this question.
Strongly Disagree
Disagree
**Agree**
Neutral
Strongly Agree
Don’t Know / Prefer not to say

**Part VII: Your Hopes for the Future of the Local Language (Transmission Intention - INT)**

Looking ahead, please express your level of hope for the transmission of the local language on a 5-point scale from (1) Very Low Hope to (5) Very Hopeful.

| Item | Statement |
| --- | --- |
| INT1 | I hope that my own children/grandchildren will be able to speak the local language fluently. |
| INT2 | I hope that in the future, local school education will place more emphasis on teaching the local language. |
| INT3 | I hope that 10 years from now, the local language will be used more widely in public spaces than it is today. |

**Part VIII: Open-Ended Questions**

**1. In your opinion, what is the most effective way to make the local language “cool” (i.e., popular) among young people?**
____________________________________________________

**2. If you were to describe the unique charm of your local language to a friend from out of town, what would you say?**
____________________________________________________

This is the end of the survey. Your participation has contributed valuable data to the study of language and culture. Thank you again for your sincere support!

**Appendix B**

**Table B1.** Normality Assessment for Core Constructs

| Construct | N | Mean | SD | Skewness | Kurtosis | Shapiro-Wilk (W) | Shapiro-Wilk (p) |
| --- | --- | --- | --- | --- | --- | --- | --- |
| INT (Transmission Intention) | 390 | 4.13 | 0.81 | -1.32 | 1.65 | 0.91 | < .001 |
| ATT (Attitude) | 390 | 4.08 | 0.70 | -1.21 | 1.76 | 0.93 | < .001 |
| V (Traditional Vitality) | 390 | 3.80 | 0.69 | -0.65 | 0.49 | 0.95 | < .001 |
| DV (Digital Vitality) | 390 | 3.58 | 0.83 | -0.55 | 0.42 | 0.96 | < .001 |
| CE (Community Engagement) | 390 | 3.18 | 0.85 | -0.52 | 0.31 | 0.96 | < .001 |
| P (Perceived Policy) | 390 | 3.01 | 0.96 | -0.15 | -0.32 | 0.98 | < .001 |


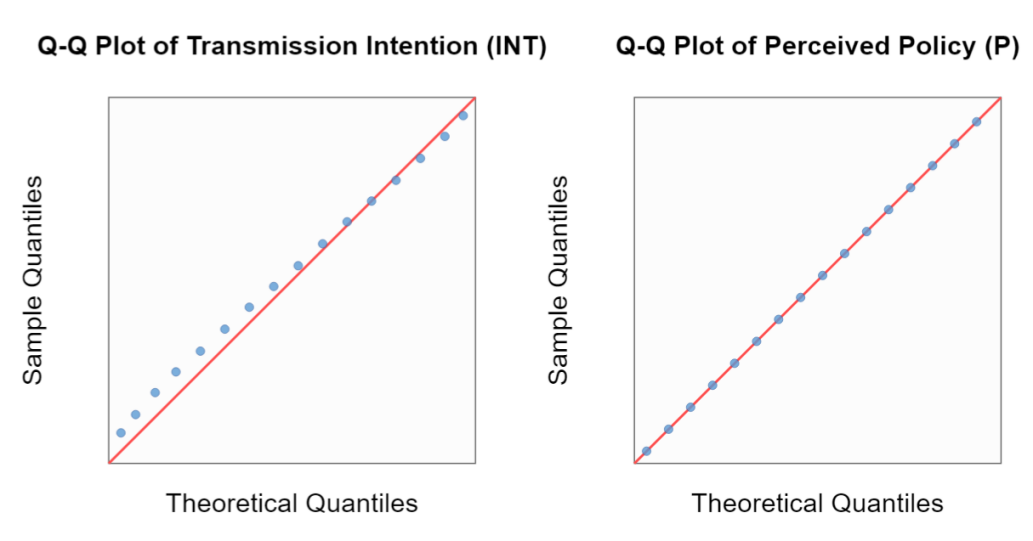


**Figure B1**.Q-Q Plots for Transmission Intention (Left) and Perceived Policy (Right). Plots for other constructs showed similar patterns of near-normality.
