# Supplementary material for: The power of perception: how perceived language policy shapes intergenerational cultural transmission intent
Source: Front Psychol. 2026 Mar 12;17:1768026. doi: 10.3389/fpsyg.2026.1768026 (PMC13017853; doi:10.3389/fpsyg.2026.1768026)
Supplement: Supplementary file 2 [file Data_Sheet_2.zip › Codebook_Anonymized.docx]

# **Data Codebook**

**For the study: Vitality, Social Support, and Transmission of Local Language**

#### **I. General Coding Rules**

All Likert scale items are coded on a 1-5 scale.

“Don’t Know” or “Not Applicable” responses are coded as 99 (or 6 in raw data) and treated as missing values (NaN) in analysis.

**Anonymity Note:** To protect participant privacy, the City column and all Open-ended text responses have been removed from the shared dataset.

#### **II. Variable Description**

| Variable Name | Item Description | Coding Rule / Scale |
| --- | --- | --- |
| Part I: Demographics |  |  |
| ID | Participant ID | Numeric (1-390) |
| City | City of residence | *[Removed for Anonymity]* |
| Age | Age Group | 1=≤18; 2=19-30; 3=31-45; 4=46-60; 5=≥61 |
| Proficiency | Local language proficiency | 1=Native-like; 2=Fluent; 3=Simple conversation; 4=Understand only; 5=Cannot speak/understand |
| FamilyLang | Family language environment | 1=Local Language; 2=Mixed; 3=Mandarin; 4=Other |
| Part II: Attitude (ATT) | *1=Strongly Disagree … 5=Strongly Agree* |  |
| ATT1 | Helpful for jobs/business | 1-5 Scale |
| ATT2 | Economic benefits for tourism | 1-5 Scale |
| ATT3 | Helps social rapports | 1-5 Scale |
| ATT4 | Identity symbol | 1-5 Scale |
| ATT5 | Confidence in speaking | 1-5 Scale |
| Part III: Traditional Vitality (V) | *1=Very Uncommon … 5=Very Common* |  |
| V1 | Intergenerational transmission | 1-5 Scale |
| V2 | Used among children/teens | 1-5 Scale |
| V3 | Used in daily markets | 1-5 Scale |
| V4 | Used in festivals/rituals | 1-5 Scale |
| V5 | Presence in local TV/Radio | 1-5 Scale |
| V6 | Availability of books/materials | 1-5 Scale |
| Part IV: Policy Support (P) | *1=Very Weak … 5=Very Strong* |  |
| P1 | Public transportation | 1-5 Scale (99/6 = Missing) |
| P2 | Public service windows | 1-5 Scale (99/6 = Missing) |
| P3 | Classes in schools | 1-5 Scale (99/6 = Missing) |
| P4 | Funding for local arts | 1-5 Scale (99/6 = Missing) |
| P5 | Intangible Heritage protection | 1-5 Scale (99/6 = Missing) |
| Part V: Community Engagement (CE) | *1=Very Low … 5=Very High* |  |
| CE1 | Community language corners | 1-5 Scale |
| CE2 | Volunteer organizations | 1-5 Scale |
| CE3 | Artistic creation usage | 1-5 Scale |
| CE4 | Commercial/Brand usage | 1-5 Scale |
| CE5 | Creative products (cultural goods) | 1-5 Scale |
| Part VI: Digital Vitality (DV) | *1=Very Uncommon … 5=Very Common* |  |
| DV1 | Social media slang/puns | 1-5 Scale |
| DV2 | Ease of typing (Input methods) | 1-5 Scale |
| DV3 | Short videos/Livestreaming | 1-5 Scale |
| DV4 | Sticker packs/Online communities | 1-5 Scale |
| Attention Check |  |  |
| AC1 | Attention Check Item | 3 = Pass (All others = Fail) |
| Part VII: Transmission Intention (INT) | *1=Very Low Hope … 5=Very Hopeful* |  |
| INT1 | Hope children speak fluently | 1-5 Scale |
| INT2 | Hope schools value it more | 1-5 Scale |
| INT3 | Hope public usage increases | 1-5 Scale |
| **Part VIII: Open-Ended Questions** | Qualitative Data |  |
| **Open_Cool1 / Open_Cool2** | Suggestions & Descriptions | [Text responses removed from this dataset to protect anonymity and strictly limit scope to quantitative analysis] |
